# A Novel Cold-Active Lipase from *Candida albicans*: Cloning, Expression and Characterization of the Recombinant Enzyme

**DOI:** 10.3390/ijms12063950

**Published:** 2011-06-14

**Authors:** Dong-Ming Lan, Ning Yang, Wen-Kai Wang, Yan-Fei Shen, Bo Yang, Yong-Hua Wang

**Affiliations:** 1 School of Bioscience and Bioengineering, South China University of Technology, Guangzhou 510006, China; E-Mails: landongming@yanhoo.com.cn (D.-M.L.); wshyn091011@gmail.com (N.Y.); breezekai@yahoo.com.cn (W.-K.W.); petsyf@126.com (Y.-F.S.); 2 Key Lab of Fermentation and Enzyme Engineering, College of Light Industry and Food Sciences, South China University of Technology, Guangzhou 510641, China

**Keywords:** *Candida albicans*, cold-active lipase, *Pichia pastoris* expression, enzyme purification

## Abstract

A novel lipase gene *lip5* from the yeast *Candida albicans* was cloned and sequenced. Alignment of amino acid sequences revealed that 86–34% identity exists with lipases from other *Candida* species. The lipase and its mutants were expressed in the yeast *Pichia pastoris*, where alternative codon usage caused the mistranslation of 154-Ser and 293-Ser as leucine. 154-Ser to leucine resulted in loss of expression of Lip5, and 293-Ser to leucine caused a marked reduction in the lipase activity. Lip5-DM, which has double mutations that revert 154 and 293 to serine residues, showed good lipase activity, and was overexpressed and purified by (NH_4_)_2_SO_4_ precipitation and ion-exchange chromatography. The pure Lip5-DM was stable at low temperatures ranging from 15–35 °C and pH 5–9, with the optimal conditions being 15–25 °C and pH 5–6. The activation energy of recombinant lipase was 8.5 Kcal/mol between 5 and 25 °C, suggesting that Lip5-DM was a cold–active lipase. Its activity was found to increase in the presence of Zn^2+^, but it was strongly inhibited by Fe^2+^, Fe^3+^, Hg^2+^ and some surfactants. In addition, the Lip5-DM could not tolerate water-miscible organic solvents. Lip5-DM exhibited a preference for the short-and medium-chain length *p*-nitrophenyl (C4 and C8 acyl group) esters rather than the long chain length *p*-nitrophenyl esters (C12, C16 and C18 acyl group) with highest activity observed with the C8 derivatives. The recombinant enzyme displayed activity toward triacylglycerols, such as olive oil and safflower oil.

## 1. Introduction

Lipases (EC 3.1.1.3) are industrially important lipolytic enzymes which are widely used as biocatalysts in biotechnological applications [1]. They are able to catalyze both the hydrolysis and synthesis of ester bonds of triacylglyceride. Lipases can be used to enrich polyunsaturated fatty acids from crude fish oil [2], modify chemical structures of oil and fats [3], and develop food flavors [4]. The special properties of lipases, such as high regional and stereo selectivity, stability in organic solvent, make them very valuable in the production of optically active compounds and esters [5]. In addition, the temperature stability of lipases is one of most important characteristics for use in industry. Recently, psychrophilic lipases have attracted more attention because of their potential in terms of lower energy costs, therapeutic and detergent applications and lower microbial contamination in industrial processes [6]. Until now, some psychrophilic microorganisms, including *Pseudomonas* sp., *Aeromonas hydrophila*, *Pseudoalteromonas* sp. and *Candida antarctica* have been found to produce cold-active lipases, most of which were produced by growth of the wild type stains [7]. However, low productivity and too many protein contaminants from culture medium were the major obstacles to obtain pure lipases for further research on their enzymatic characteristics. Recombinant expression of lipases is a good way to solve the problem. Ryu *et al*. expressed M37 lipase from a *photobacterium* strain in *E. coli*, and the recombinant lipase displayed maximum activity at 25 °C and maintained its activity at a low temperature range (5–25 °C) with an activation energy (*E*_a_) of 2.07 kcal/mol [8]*. EML1* lipase gene was isolated from deep-sea sediment metagenome, and the recombinant EML1 lipase was expressed in *E. coli* exhibited maximum activity at 25 °C and still maintained more than 50% activity at 5 °C [9]. Parra *et al*. have reported that a lipase from *Psychrobacter sp* showed highest enzymatic activity at 20 °C and pH 8.0, and the activation energy was 5.5 kcal/mol when the temperature was between 5 and 20 °C [10]. Some research has been performed to disclose the structural perpetrates of lipases in order to know its catalytic mechanism at low temperature. It has been reported that the possible features of cold-active lipases contain conformations such as a small hydrophobic core, a very small number of salt bridges and of aromatic-aromatic interactions, a reduced number of disulphide bridges and of prolines in loop structures, lower number of ion pairs and weakening of charge-dipole interactions in α-helices [11]. Cold-active lipases from different microorganisms have so far been found to be useful for industrial application; further discovery of new lipases with commercially useful properties still attracts a great deal of interest. Different kinds of lipases (Lip1-Lip10), which show high sequence identities in gene sequences, have been found in yeast *Candida albicans* (*C. albicans*) [12], but the characterization of most of the lipases has not been reported yet. In this study, a “novel” lipase (Lip5) from *C. albicans* was cloned and expressed in *Pichia pastoris* (*P. pastoris*). The mutants of *lip*5 which mutated CTG to TCT codon was also investigated because *C. albicans* has a special protein translation system. Tulte *et al*. reported that the yeast *C. albicans* encoded a unique seryl-tRNACAG that should decode the leucine codon CUG as serine instead of leucine [13]. The Lip5 and its mutant proteins were purified by ammonium sulfate precipitation followed by ion-exchange chromatography. The characterization, such as effect of pH, temperature, and ion on the enzymatic activity, was explored. Studies of enzyme kinetics and substance specificity on different fatty acids chains were also performed. This study firstly showed that the Lip5 was a cold-active lipase from yeast *C. albicans*, which contradicts the report that the cold-active enzyme is usually from the cold environment such as polar region or deep sea.

## 2. Results and Discussion

### 2.1. Cloning of *lip5-wt* Gene and Mutants from *C. albicans*

Sequence analysis of *lip5-wt* from *C. albicans* showed no introns in the *lip5-wt* gene sequence, and a putative N terminus signal sequence includes 14 amino acid residues, which exports the Lip5 outside the cells. *lip5-wt* gene with the deleted signal sequence was cloned from *C. albicans* genome DNA and sequenced. It was found that two non-universal Ser codons (CTG) exist in the *lip5-wt* gene, located in amino acid site 154 and 293 respectively. Triplet codon CTG was usually mistranslated into leucine in other organisms instead of serine [14]. To investigate the influence of codon CTG on the expression and characterization of *lip5-wt*, three mutants, which are *lip5-m_154_*, *lip5*-*m_293_* and *lip5*-*dm*, were constructed. *lip5*-*m_154_*, *lip5*-*m_293_* indicates that the corresponding gene codon of 154 and 293 amino acid were modified as TCT, respectively. *lip5*-*dm* was the double mutant at above two sites. The primers for gene mutation were listed in Table 1.

### 2.2. Amino Acid Sequence Analysis

**Figure 1:**
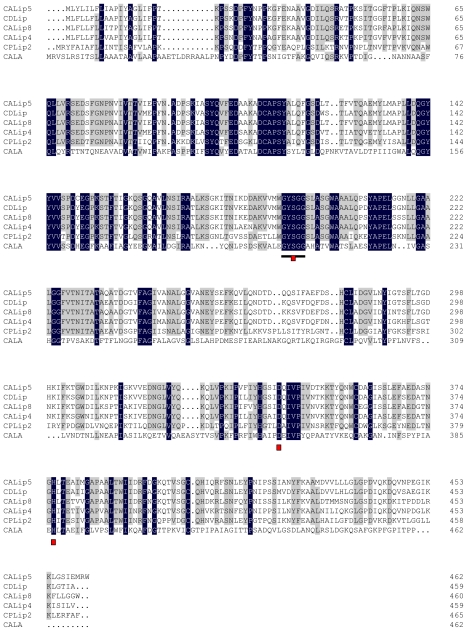
Multiple alignment of Lip5-DM (CALip5) amino acids sequence (HM581681) with those of lipases from other *Candida* species. The lipase sequence included CDLip (putative lipase from *Candidas dubliniensis* CD36, XP_002421466), CALip8 (lipase 8 from *Candidas albican* SC5314, AAF69523), CALip4 (lipase 4 from *Candidas albican* SC5314, AAF69521), CPLip2 (lipase 2 from *Candida parapsilosis*, CAC86400) and CALA (lipase A from *Candida antarctica*, 3GUU_A). Gray shading reflects the degree of sequence conservation. The conserved pentapeptide (Gly-Xaa-Ser-Xaa-Gly) is underlined. The putative active sites on Lip5 sequence are marked with red rectangles.

A search of Genbank using the deduced Lip5 amino acid sequence (HM581681) as a query by Blastp program revealed that Lip5 shared significant sequence identity with those of lipases from other *Candidas* species. Lip5 has 87%, 84%, 81%, 58% and 34% amino acid sequence identity with those of lipases from *Candida dubliniensis* (CDLip), *Candida albiacans* (CALip8 and CALip4) [15,16], *Candida parapsilosis* (CPLip2) [17] and *Candida Antarctica* (CALA) [18], respectively. It was also found that Lip5 shows low sequence identity with those of known lipases from psychrophilic organisms, including *Photobacterium* sp. M37 [8], *Pseudomonas* sp. 7323 [19], *Pseudomonas fragi* [20] and *Psychrobacter* sp. 2–17 [21] (data not shown). In addition, sequence alignment showed that all the lipase sequences contained a highly conserved pentapeptide GYSGG (Figure 1), which is consistent with the GXSXG motif found in the active site of most of the lipolytic enzyme [22]. Based on the alignment results, the putative catalytic triad of Lip5 is composed of Ser-194, Asp-343 and His-376 (Figure 1).

### 2.3. Expression of *lip5-wt* and Mutants in *P. pastoris*

**Figure 2:**
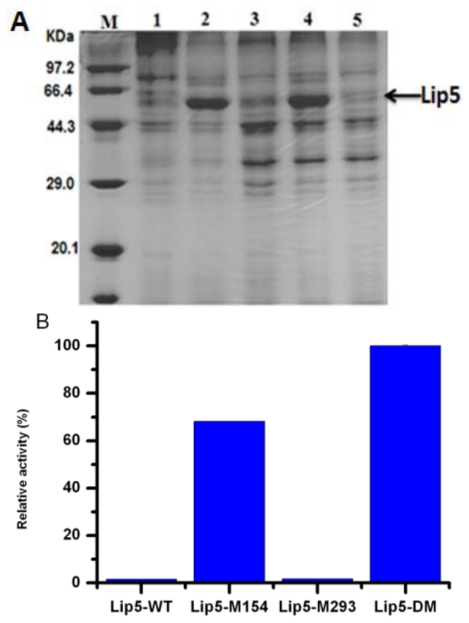
(**A**) Lane M: molecular weight marker; lane 1: Sample from wild type Lip5 expression medium; lane 2: Sample from Lip5-M154 expression medium; lane 3, sample from Lip5-M293 expression medium; lane 4: Sample Lip5-DM expression medium; lane 5: sample from transformant harboring pGAPZαA expression medium; (**B**) The activity of Lip5-WT and mutants in the culture supernatant were measured spectrophotometrically with *p*-NP**-**caprylate as substrate at 25 °C and pH 6.0. Activities are displayed as percentages of the maximum activity (Lip5-DM, 4000 U/L).

Lipases with different gene sequences, which included *lip5-wt*, *lip5-m_154_*, *lip5-m_293_* and *lip5-dm*, have been expressed secretively in *P. pastoris* under the control of the glyceraldehydes-3-phosphate dehydrogenase (GAP) constitutive promoter, and their activities were investigated and the results were shown in Figure 2. Lip5-M_154_ and Lip5-DM was expressed at high level with a molecular weight of about 50 kDa (Figure 2A), however, no obvious expression was observed from Lip5-WT and Lip5-M_293_. It suggested that mistranslation of 154-Ser to leucine resulted in loss of expression of Lip5-WT, and that 154-Ser might either play an important role in the stability of the Lip5-WT, or have a negative effect on the expression of Lip5-WT. Roustan *et al*. have reported that a lipase from *C. albicans* (CaLip4) could be overproduced in *Saccharomyces cerevisiae* only after site mutation of its CUG codon (159-Ser) into a universal one [15]. Lip5-DM, which has double mutagenesis of 154 and 293 amino acids, showed better activity than Lip5-M_154_ (Figure 2B). The specific activity of pure Lip5-DM was also higher than that of Lip5-M_154_ (data not shown). It indicated that 293-Ser might be a critical amino acid which contributes to the enzymatic activity. Similar results were found for lipases from *Candida rugosa*, which required replacement of all the non-universal condons by universal serine codons with site-directed mutagenesis or whole gene synthesis methods to obtain functional lipase in universal host such as *P. pastoris* and *E. coli* [23–27].

### 2.4. Purification of Recombinant Lip5-DM

**Figure 3:**
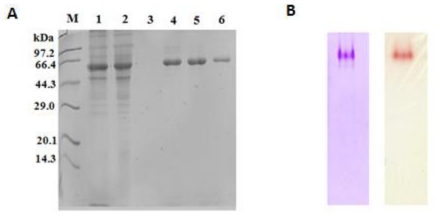
Purification of Lip5-DM from the culture supernatant (**A**) and activity staining (**B**) A Lane M: molecular weight marker; lane 1: Culture supernatant; lane 2: Concentrated supernatant after (NH_4_)_2_SO_4_ precipitation; lane 3: Fraction sample eluted from column by 100 mM NaCl; lane 4–6: Fraction samples eluted from Q Sepharose™ Fast Flow by 200 mM NaCl. B. Zymogram analysis of purified Lip5-DM (1.4 μg) was performed with β-naphtyl acetate as substrate. The gels were stained by Coomassie Brilliant Blue (left) and (right) Fast Blue RR.

The recombinant Lip5-DM was separated from the culture media by two purification steps according to the method described in the experimental section. The crude lipase protein was precipitated with the increment of mass fraction of (NH_4_)_2_SO_4_, and the maximum protein precipitation was obtained when the mass fraction of (NH_4_)_2_SO_4_ was more than 70%. The crude lipase, which was precipitated completely by 80% of (NH_4_)_2_SO_4_, was dissolved in Tris-HCl buffer (20 mM, pH 8.0), and was purified further by anion-exchange chromatography. The recombinant Lip5-DM could be eluted when the concentration of NaCl increased to 200 mM, and the fractionated eluant was analyzed by SDS-PAGE (Figure 3A). Zymogram analysis shows that the Lip5-DM was purified to homogeneity (Figure 3B). The pure lipase was collected for further analysis of enzymatic characterization.

### 2.5. Effect of Temperature on the Lipase Activity and Stability

**Figure 4:**
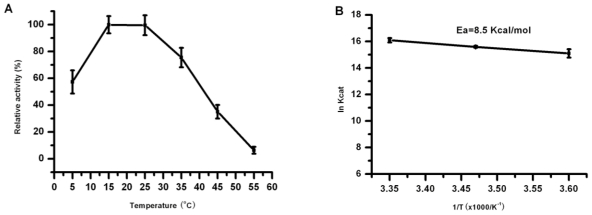
Effects of temperature on Lip5-DM activity: (**A**) The purified Lip5-DM was assayed at different temperatures. Activities are displayed as percentages of the maximum activity (31.27 U/mg). Values are means ± SD from three independent experiments; (**B**) To determine the activation energy, the logarithm of the *K*_cat_ values was plotted against the reciprocal of absolute temperature (*T*). The values shown are activation energy calculated from the Arrhenius plot.

**Figure 5:**
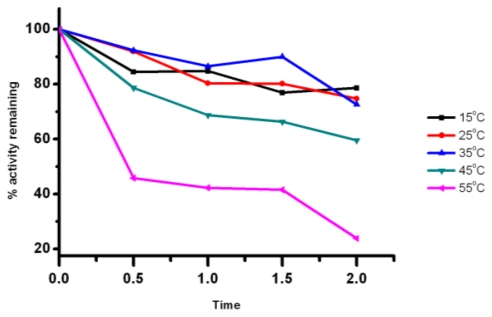
Effect of temperature on the recombinant lipase thermostability. The enzyme was assayed after incubation in a range of temperatures (15–55 °C) for 2 h. Activities are displayed as percentages of the initial activity (31.27 U/mg).

To determine the optimal temperature of the recombinant lipase, lipolytic activity of Lip5-DM to *p*-NP-caprylate was measured under different temperatures (5–55 °C). The relative activities at various temperatures are shown in Figure 4A, taking the activity at 15 °C as 100%. It is interesting to observe that the recombinant lipase displayed the highest activity at 15 and 25 °C, and maintained more than 70% of the maximum activity at 35 °C. Even at a lower temperature of 5 °C, the recombinant lipase still retained more than 50% of the maximal activity (Figure 4A). The optimum temperatures for Lip5 was lower than those of lipase CALip4, CPLip2 and CALA, which were 50–60 °C [15], 50 °C [17] and 50–70 °C [28], respectively. The Lip5-DM exhibited high hydrolytic activity at low temperature, and this property was similar to that of cold active lipase from psychrophilic organisms with high activity in a temperature range of 0–30 °C [6,29]. The reactions catalyzed by the enzymes derived from cold-adapted organisms are usually lower than those catalyzed by the corresponding enzymes from their mesophilic counterparts [30]. Therefore, we further determined the activation energy for hydrolysis of *p*-NP-caprylate catalyzed by Lip5-DM. The value of activation energy was about 8.5 kcal/mol in the temperature range from 5 to 25 °C (Figure 4B). This value was lower than that of a cold-adapted lipase LipP from an Alaskan Psychrotroph, *Pseudomonas* sp. Strain B11-1, with an activation energy of 11.2 kcal/mol [31] and it was higher than that of cold active lipases from *Psychrobacter* sp. and a deep-sea sediment metagenome, with activation energy of 5.5 kcal/mol for MBP-lipase and 3.28 kcal/mol for rEML1, respectively [9,10]. Thermostability of Lip5-DM was also investigated by measuring the residual activities during incubation at various temperatures for 2 h at pH 6.0. The Lip5-DM showed good thermostability over a range of temperature from 15 °C to 45 °C, with more than 60% of maximal activity retained after 2 h incubation (Figure 5). However, the stability of recombinant lipase decreased at 55 °C, with 23.86% residual activity after incubation for 2 h. These thermal properties were similar to that displayed by a cold active lipase from *Aspergillus nidulans*, which sharply lost its activity when the temperature exceeded 40 °C [32].

### 2.6. Effect of pH on Lip5-DM Activity and Stability

**Figure 6:**
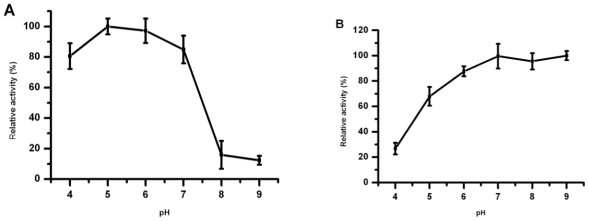
Influence of pH on Lip5-DM activity (**A**) and stability (**B**). The activity of purified Lip5-DM was measured at various pHs from 4.0 to 9.0. For the stability study, the lipase enzyme was investigated after incubation in a range of pH (4.0–9.0) for 12 h. Activities are shown as percentages of the maximum activity (32.17 U/mg). Values are means ± SD from three independent experiments.

To investigate the influence of pH on Lip5-DM, the activity of lipase was measured at various pHs. The recombinant lipase was active in a pH range of 5.0–7.0, with the maximal activity at pH 5.0 and 6.0 (Figure 6A). The pH stability of lipase was explored by pre-incubating Lip5-DM in buffer with different pH at 4 °C for 12 h. The results are shown in Figure 6B. The Lip5-DM activity exhibited stable within the pH range of 5–9, and it remained more than 60% of maximal activity. The recombinant lipase sharply lost its activity when incubated at pH 4.0, and only preserved 26% activity.

### 2.7. Effect of Metal Ions, Inhibitors, Detergents and Water-Miscible Solvent on the Activity of Lip5-DM

Effect of metal ions on the recombinant Lip5-DM hydrolysis activities was determined. The metal ion concentration was selected as 1 mM and 5 mM respectively. The results are shown in Table 2. Among the metals ions tested, Zn^2+^ increased the lipase activity at either low (1 mM) or high concentration (5 mM) with respect to the blank control, possibly by involving stabilizing the active conformation of the enzyme. The structural stabilizing role of Zn^2+^ on lipase from *Geobacillus stearothermophilus* has been observed by Choi *et al*. [33]. Fe^2+^, Fe^3+^ and Hg^2+^ strongly inhibit the lipase activity, and the residual activities of lipase were 20%, 35% and 13% respectively when their concentrations were 5 mM. A similar inhibitory effect of these ions on the activity of microbial lipases has been reported by other authors [34–36]. The activity of lipase was inhibited by Hg^2+^, suggesting that the thiol-group harboring amino acids might be involved in activation of the lipase. Ethylenediaminetetraacetic acid (EDTA) inhibited the activity of lipase by about 38% (1 mM), which showed that certain metal ions might contribute to the catalysis mechanism of lipase. Moreover, Phenylmethanesulfonyl fluoride (PMSF) also decreased the recombinant lipase activity, indicating that the Ser residues played an important role in the biocatalysis function of the lipase. Surfactants such as Tween 20, Tween 80 and TritonX-100 showed inhibitory effect in the lipase activity (Table 3). Sodium dodecyl sulfate (SDS) sharply decreased the lipase activity by 85% with a low concentration of 0.1%. Methanol, ethanol, and acetone showed negative effect on the lipase activity, and the residual activities of Lip5-DM remained at 48%, 24% and 44% respectively after incubation for 1 h. Lipase activity was almost lost in the presence of isopropanol alcohol. These properties differ from those displayed by lipase from *Fusarium heterosporum*, which was activated by pre-incubation in organic solvent [37].

### 2.8. Substrate Specificity of Lip5-DM

**Figure 7:**
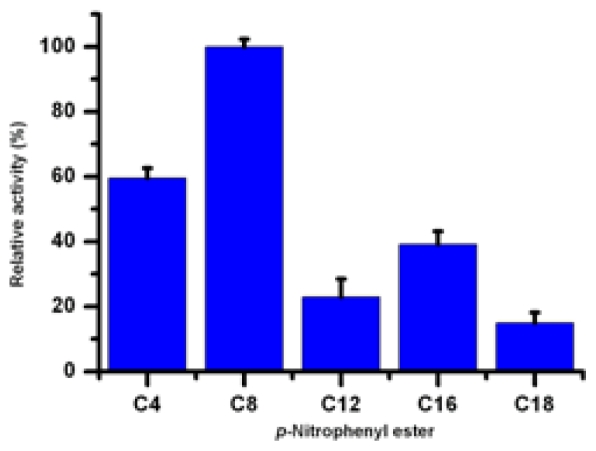
Relative activity of Lip5-DM on various *p*-nitrophenyl esters with different chain lengths. Activities on each substrate are expressed as the percentage of *p*-NP-caprylate (31.27 U/mg). Values are means ± SD from three independent experiments.

A series of *p*-NP esters with a variety of carbon chain lengths was employed to determine the substrate specificity of recombinant Lip5-DM. It was observed that Lip5-DM exhibited a preference for the short and medium chain length *p-*NP (C4 and C8 acyl group) esters rather than the long chain length *p-*NP esters (C12, C16 and C18 acyl group), with relative activity of 100, 59, 39, 22 and 14% for the C8, C4, C16, C12 and C18 derivatives, respectively (Figure 7). Similar substrate preference property was found for CALip4, which displays maximum hydrolysis activity to the C6 and C8 *p*-NP derivatives [15]. The kinetic constant were determined and summarized in Table 4. The highest *K_cat_* value was observed with *p-*NP-caprylate (C8), whereas *p-*NP-laurate (C12) showed the lowest *K_cat_* value. Among the *p*-nitrophenyl esters tested, *p-*NP-caprylate (C8) was observed to have the highest specificity constant (*K_cat_*/*K_m_*), and the *K_m_* and *K_cat_* values were 0.27 mM and 55.12 S^−1^, respectively. Therefore, *p-*NP-caprylate (C8) was the most suitable substrate for the recombinant enzyme.

To determine the lipase activity of recombinant Lip5-DM, triacylglycerols such as olive oil and safflower oil were hydrolyzed by recombinant enzyme, and the hydrolysis activities were 3200 U/L and 3800 U/L, respectively. This showed that the Lip5-DM was a lipase and displayed lipolytic activity to lipid.

## 3. Experimental Section

### 3.1. Strains, Plasmids and Materials

Strain *C. albicans* ATCC 10231 was collected from Guangdong Microbial Culture Collection Center. *E. coli* DH5α, *P. pastoris* X33 (Invitrogen), plasmid pBluescript SK vector (Stratagene) and pGAPZαA (Invitrogen) were used for gene cloning and protein expression. The substrates (*p*-nitrophenyl (*p*NP) esters derivates) were purchased from Sigma. Other reagents and solvents were of analytical grade.

### 3.2. Cloning of *lip5-wt* and Sequence Analysis

*C. albicans* genomic DNA was prepared using a fungal genome extraction kit (Omega) according to the manufacturer’s instruction. PCR was performed to amplify the *lip5-wt* gene sequence (without the signal peptide) using *C. albicans* genome DNA as template. The primers were Lip5-FP and Lip5-RP (Table 1). The amplified fragments were inserted into pBluescript vector, resulting plasmid pBluescript-*lip5-wt*, and completely sequenced. The nucleotide sequence of *lip5* reported in this work has been deposited in the GenBank under the accession number HM581681. Multiple alignments of amino acid sequences of *lip5-wt* with other lipases from other *Candida* species were performed by the program ClustalW2 (http://www.ebi.ac.uk/Tools/clustalw2).

### 3.3. Construction of the Mutant lip5 Gene Variants

Triplet codon CTG in the *lip5-wt* gene sequence, which is translated into serine in *C. albicans*, was mutated into TCT by overlap extension PCR method. The primers are shown in Table 1. Three DNA fragments (*lip5-A*, *lip5-B* and *lip5-C*) were produced by PCR using pBluescript-*lip5-wt* as template. *lip5-dm*, which has double mutant, was produced using the mixture of the three purified DNA fragments of (*lip5-A*, *lip5-B* and *lip5-C*) as template with primer Lip5-FP and Lip5-RP. To obtain *lip5*-*m_154_* gene containing only a single mutation in site 154 amino acid, PCR was performed with primer Lip5-CTG_154_-FP and Lip5-RP using pBluescript-*Lip5*-*wt* as template to yield DNA fragment *lip5-BC*. Then fusion of *lip5-A* and *lip5-*BC was performed to generate *lip5*-*m_154_* with primers lip5*-*FP and lip5*-*RP. Similarly, *lip5*-*AB* was produced by PCR with primers *lip5-*FP and lip5-CTG_293_-RP. And then, *lip5*-*m_293_* gene was obtained by fusion of DNA fragment *lip5*-*AB* and *lip5*-*C.* All resulting DNA fragments *lip5*-*dm*, *lip5*-*m_154_* and *lip5*-*m_293_* were inserted into pBluescript vector at the site of *Kpn* I and *Xho* I to produce pBluescript-*lip5*-*dm*, pBluescript-*lip5*-*m_154_* and pBluescript-*lip5*-*m_293_*, respectively. The plasmids containing targeted genes were fully sequenced. And then, subcloning of these genes to the expression vector pGAPZαA was performed to generate vector pGAPZαA-*lip5-dm*, pGAPZαA-*lip5-m_154_* and pGAPZαA-*lip5-m_293_*.

### 3.4. Expression of *lip5-wt*, *lip5-m*_154_, *lip5-m*_293_ and *lip5-dm*

Vectors pGAPZαA-*lip5*-*dm*, pGAPZαA-*lip5*-*m*_154_, pGAPZαA-*lip5*-*m*_293_ and pGAPZαA were linearized by *Avr*II digestion. The purified digestion products were transformed into *P. pastoris* X-33 by electroporation, and the transformants were screened on YPD plates (1% yeast extract, 2% peptone, 2% dextrose and 2% agar) containing 100 μg/mL Zeocin. The transformed strains were grown in YPD liquid medium for protein expression. The single transformed colony was picked into 3 mL YPD medium containing 100 μg/mL Zeocin in a tube and grown at 30 °C and 200 rpm until the OD_600_ reached 2–6. Then the seed culture was inoculated into 100 mL YPD and cultured at 30 °C for 72 h.

### 3.5. Purification of Recombinant Lip5-DM

All the purification steps were performed at 4 °C. The supernatant was clarified by centrifugation (12,000 × g, 4 min, 4 °C) to remove cells. The crude enzyme solution was concentrated by ammonium sulfate precipitation (50–80%) and the precipitated fractions were collected by centrifugation (12,000 × g, 30 min), and then the crude lipases precipitation was dissolved with dilution buffer (20 mM Tris-HCl, pH 8.0) for further purification by anion-exchange chromatography with a Q Sepharose™ Fast Flow column (0.8 × 15 cm, GE Healthcare). The samples were applied onto the column which was pre-equilibrated with 20 mM Tris-HCl (pH 8.0), and then washed with the buffer (20 mM Tris-HCl, pH 8.0) and the buffer (20 mM Tris-HCl, 100 mM NaCl, pH 8.0) respectively. The recombinant Lip5-DM was eluted with the buffer (20 mM Tris-HCl, 200 mM NaCl, pH 8.0) at a flow rate of 3 mL/min, and fractionated eluant from the column were analyzed by SDS-PAGE.

### 3.6. Lipase Activity and Protein Analysis

Esterase activity was measured by spectrophotometric method using *p*-nitrophenyl caprylate (*p*-NP**-**caprylate) as substrate. The reaction mixture consisted of 100 mM Na_2_HPO_4_-NaH_2_PO_4_ buffer (pH 6.0), 2 mM *p*-NP**-**caprylate and an appropriate amount of the lipase. The reaction was carried out at 25 °C for 5 min. The amount of released *p*-nitrophenol was quantified by its absorbance at 405 nm by a spectrophotometer. One unit of activity was defined as the amount of enzyme needed to release 1 μmol of *p*-nitrophenol per minute under the assay conditions.

Lipase activity was quantified at pH 6.0 by free fatty acid titration with 20 mM NaOH after incubated for 10 min at 25 °C in a vessel. The assay mixture consisted of 4 mL 100 mM Na_2_HPO_4_-NaH_2_PO_4_ buffer, 5 mL emulsified olive oil or sufflower oil and 1ml enzyme solution. One unit of lipase activity was defined as the amount of enzyme releasing 1μmol of free fatty acids per minute. Protein samples were mixed with loading buffer and heated to 95 °C for 5 min and loaded onto 12% SDS-PAGE gels. Proteins were stained by Coomassie blue as instructions. Protein concentration was determined using a BCA Protein Assay Kit (Sangon, Shanghai, China) according to the manufacturer’s instruction.

### 3.7. Zymogram Analysis

Zymogram analysis was done by running the purified lipase sample on a 12% native PAGE gel. After electrophoresis, the gel was incubated for 30 min in 50 mM phosphate buffer (pH 7.2) containing 1% β-naphthyl acetate at room temperature. After addition of 0.5% Fast Blue RR solution, enzyme activity could be detected by the appearance of brown colored bands in the gel.

### 3.8. Effect of Temperature on the Lip5-DM Activity and Stability

Effect of temperature on the lipase activity was determined by measuring the hydrolytic activity at different temperatures in 100 mM Na_2_HPO_4_-NaH_2_PO_4_ buffer (pH 6.0). The temperature was set as 5 °C, 15 °C, 25 °C, 35 °C, 45 °C and 55 °C. For investigation of the thermostability, the purified lipase was incubated at different temperature for 2 h, and activity measurements were performed every 30 min for each temperature.

### 3.9. Effect of pH on the Lip5-DM Activity and Stability

The optimum pH was investigated by measuring the hydrolytic activity of lipase at various pH value. The pH was set as 4, 5, 6, 7, 8, 9. The buffer was used as following: 100 mM sodium acetate (pH 4.0, pH 5.0), 0.1 M Na_2_HPO_4_-NaH_2_PO_4_ (pH 6.0, pH 7.0), 50 mM Tris-HCl (pH 8.0) and 50 mM Gly-NaOH (pH 9.0). The reaction was performed at 25 °C. To analyze pH stability of lipase, the pure Lip5-DM was pre-incubated in buffers within the different pH value at 4 °C for 12 h. Then the residual activity was analyzed.

### 3.10. Effect of Metal Ions, Inhibitor, Surfactants and Water-Miscible Solvent on the Lip5-DM Activity

The influence of various chemicals, surfactants, inhibitors and water-miscible solvent on the hydrolytic activity was determined by detecting the residual activity at 25 °C after incubating the pure Lip5-DM in 100 mM Na_2_HPO_4_-NaH_2_PO_4_ (pH 6.0) buffer containing each of various metal ions (1 mM and 5 mM), each of surfactants (0.1% v/v) and each of water-miscible solvents (30% v/v) at 4 °C for 1 h. The control was performed without metal ions, detergents and water-miscible solvents. The metal ions include ZnSO_4_, CuSO_4_, MgSO_4_, FeCl_3_, CaCl_2_, MnSO_4_, HgCl_2_, LiCl, FeSO_4_, NiCl_2_, BaCl_2_, CoCl_2_. Inhibitors include Ethylenediaminetetraacetic acid (EDTA) and phenylmethylsulfonyl fluoride (PMSF). The detergents include Tween 20, Tween 80, Triton X-100 and SDS. The water-miscible solvents include methanol, ethanol, acetone and isopropanol alcohol.

### 3.11. Substrate Specificity

The substrate specificity of Lip5-DM was investigated using *p*-nitrophenyl fatty acid esters with various acyl chain lengths. The substrates are as following: *p*-nitrophenyl butyrate (*p*-NP**-**butyrate, C4), *p*-nitrophenyl caprylate (*p*-NP**-**caprylate, C8), *p*-nitrophenyl laurate (*p*-NP**-**laurate, C12), *p*-nitrophenyl palmitate (*p*-NP**-**palmitate, C16) and *p*-nitrophenyl stearate (*p*-NP**-**stearate, C18) were used. Determination of kinetic constants and activation energy Kinetic constants of Lip5-DM were determined by using *p*-NP esters as substrate. The initial rate of *p*-NP esters hydrolysis was measured at various substrate concentrations over the range 0.125 mM to 5 mM. The reaction was performed at 5, 15 and 25 °C according to the procedure described above. The *V_max_* and *K_m_* values were determined by Lineweaver-Burk plots method. *K_cat_* was calculated subsequently with enzyme concentration. The activation energy (*E*_a_) was determined by using the slope of the Arrhenius plot and Arrhenius equation.

## 4. Conclusions

A new cold-active lipase from the yeast *C. albicans* showed low sequence identity with those of lipases from psychrophilic organisms. Codon CTG have been mistranslated into leucine instead of serine in the *P. pastoris*, and serine is an important amino acid residue for the lipase expression and activity. Pure Lip5-DM showed high activity at low temperatures (5–35 °C) and broad pH ranges (5.0–9.0), with an activation energy of 8.5 kcal/mol. The activity of lipase increased in the presence of Zn^2+^. Surfactants and water-miscible solvents decreased lipase activity. Lip5-DM exhibited a preference for the short and medium chain length *p*-NP esters rather than the long chain length *p*-NP, and high affinity to *p*-NP**-**caprylate was observed. Noteworthy is, only CALip4, which is one of ten lipases found in the *C. albicans*, has been reported on its enzymatic characterization [15]. CALip4 showed high temperature activity at 50–60 °C, and displayed a high alcoholysis activity with a range of alcohols (e.g., methanol, ethanol, propanol and isopropanol) as acyl acceptor. It is very interesting that CALip4 and Lip5 showed a huge difference in enzymatic characterization although they are both from *C. albicans* with high sequence identity. These above results indicated that this novel lipase is a new example of a cold-active lipase with different properties. In addition, this work will be of great value to overexpress the lipase from *C. albicans* efficiently with yeast *P. pastoris* as a host cell.

## Figures and Tables

**Table 1:** Primers for lip5 gene cloning and mutation.

Primers	Sequences a (5′-3′)
Lip5-FP	GGGGTACCGGCCTTATTTTCCCTACCAA
Lip5-RP	CCGCTCGAGTTATAACCACCTCATTTCAATTG
Lip5-CTG_154_-RP	GT*AGA*TTTAGGTCCTTCATAATCAG
Lip5-CTG_154_-FP	GATTATGAAGGACCTAAA**TCT**ACATTCACTATTGGTAAACAATCAGG
Lip5-CTG_293_-RP	CTTATGATCACCAGTTAAGAA*AGA*AGTACCGATATAGTTGAGCACAC
Lip5-CTG_293_-FP	**TCT**TTCTTAACTGGTGATCATAAGA

a *Kpn*I and *Xho*I restriction sites in Lip5-FP and Lip5-RP are underlined, respectively.

Nucleotides (TCT) encoding serine is in bold.

**Table 2:** Effect of various chemicals on the recombinant lipase activity a.

Reagents	Relative activity (%)	
	1 mM	5 mM
Control	100	119.22 ± 6.61
ZnSO_4_	100	115.19 ± 7.44
CuSO_4_	95.02 ± 2.61	78.35 ± 8.35
MgSO_4_	94.49 ± 7.85	97.66 ± 9.64
FeCl_3_	64.01 ± 8.18	35.11 ± 0.51
CaCl_2_	89.57 ± 8.32	72.04 ± 1.85
MnSO_4_	111.44 ± 5.85	90.66 ± 2.38
LiCl	96.56 ± 8.31	92.24 ± 4.84
Fe SO_4_	72.99 ± 4.89	20.27 ± 2.98
HgCl_2_	18.67 ± 3.04	13.19 ± 6.60
BaCl_2_	80.36 ± 6.84	77.22 ± 5.74
NiCl_2_	96.56 ± 8.31	92.24 ± 4.84
CoCl_2_	100.86 ± 9.73	89.10 ± 3.67
EDTA	71.86 ± 10.09	65.41 ± 2.58
PMSF	52.14 ± 2.63	38.54 ± 2.15

a The purified recombinant lipase was incubated in 100 mM Na_2_HPO_4_-NaH_2_PO_4_, pH 6.0 containing each chemicals at 4 °C for 1 h. Residual activities the lipase retained were measured using *p*-NP-caprylate as substrate under standard condition. Activities are shown as percentages of the control activity value (73.8 U/mg). Values are means ± SD from three independent experiments.

**Table 3:** Influence of detergents and organic solvents on the recombinant lipase activity a.

Reagents	Relative activity (%)	
	0.1% (w/v)	30% (v/v)
Control	100	100
Tween 20	82.52 ± 9.57	
Triton X-100	48.55 ± 1.57	
SDS	15.10 ± 4.72	
Tween 80	57.24 ± 4.17	
Methanol		48.23 ± 3.17
Ethanol		24.32 ± 2.42
Acetone		44.76 ± 3.59
Isopropanol		4.59 ± 1.09

a The purified recombinant lipase was incubated in 100 mM Na_2_HPO_4_-NaH_2_PO_4_, pH 6.0 containing each detergents (0.1% (w/v)) and solvents (30% (v/v)) at 4 °C for 1 hour. Residual activities were measured using *p*-NP-caprylate as substrate under standard condition. Activities are shown as percentages of the control activity value (73.8 U/mg). Values are means ± SD from three independent experiments.

**Table 4:** Kinetic parameter of Lip5 for hydrolysis of various *p*-nitrophenyl esters.

Substrate	*K_m_* (mM)	*V*_max_ (mM/min)	*K*_cat_ (s^−1^)	*K_cat_*/*K_m_* (s^−1^·mM^−1^)
*p*-NP-butyrate	0.41	1.20	54.18	131.00
*p-*NP-caprylate	0.27	1.22	55.12	203.41
*p*-NP-laurate	0.50	0.20	9.41	18.58
*p*-NP-palmitate	0.36	0.70	31.59	86.86

## References

[1] Jaeger KE, Eggert T (2002). Lipases for biotechnology. Curr. Opin. Biotechnol.

[2] Schmitt-Rozieres M, Deyris V, Comeau LC (2000). Enrichment of polyunsaturated fatty acids from sardine cannery effluents by enzymatic selective esterification. J. Am. Oil Chem. Soc.

[3] Lumor SE, Jones KC, Ashby R, Strahan GD, Kim BH, Lee GC, Shaw JF, Kays SE, Chang SW, Foglia TA, Akoh CC (2007). Synthesis and characterization of canola oil-stearic acid-based trans-free structured lipids for possible margarine application. J. Agric. Food Chem.

[4] Kheadr EE, Vuillemard JC, El-Deeb SA (2003). Impact of liposome-encapsulated enzyme cocktails on cheddar cheese ripening. Food Res. Int.

[5] Jaeger KE, Reetz MT (1998). Microbial lipases form versatile tools for biotechnology. Trends Biotechnol.

[6] Marshall CJ (1997). Cold-adapted enzymes. Trends Biotechnol.

[7] Joseph B, Ramteke PW, Thomas G (2008). Cold active microbial lipases: Some hot issues and recent developments. Biotechnol. Adv.

[8] Ryu HS, Kim HK, Choi WC, Kim MH, Park SY, Han NS, Oh TK, Lee JK (2006). New cold-adapted lipase from Photobacterium lipolyticum sp nov that is closely related to filamentous fungal lipases. Appl. Microbiol. Biotechnol.

[9] Jeon JH, Kim JT, Kim YJ, Kim HK, Lee HS, Kang SG, Kim SJ, Lee JH (2009). Cloning and characterization of a new cold-active lipase from a deep-sea sediment metagenome. Appl. Microbiol. Biotechnol.

[10] Parra LP, Reyes F, Acevedo JP, Salazar O, Andrews BA, Asenjo JA (2008). Cloning and fusion expression of a cold-active lipase from marine Antarctic origin. Enzym. Microb. Tech.

[11] Arpigny JL, Lamotte J, Gerday C (1997). Molecular adaptation to cold of an Antarctic bacterial lipase. J. Mol. Catal. B: Enzym.

[12] Hube B, Stehr F, Bossenz M, Mazur A, Kretschmar M, Schafer W (2000). Secreted lipases of Candida albicans: cloning, characterisation and expression analysis of a new gene family with at least ten members. Arch. Microbiol.

[13] Santos MAS, Keith G, Tuite MF (1993). Nonstandard Translational Events in Candida-Albicans Mediated by an Unusual Seryl-Transfer Rna with a 5′-Cag-3′ (Leucine) Anticodon. EMBO J.

[14] Ohama T, Suzuki T, Mori M, Osawa S, Ueda T, Watanabe K, Nakase T (1993). Nonuniversal decoding of the leucine codon cug in several candida species. Nucleic Acids Res.

[15] Roustan JL, Chu AR, Moulin G, Bigey F (2005). A novel lipase/acyltransferase from the yeast Candida albicans: Expression and characterisation of the recombinant enzyme. Appl. Microbiol. Biotechnol.

[16] Gacser A, Stehr F, Kroger C, Kredics L, Schafer W, Nosanchuk JD (2007). Lipase 8 affects the pathogenesis of Candida albicans. Infect. Immun.

[17] Neugnot V, Moulin G, Dubreucq E, Bigey F (2002). The lipase/acyltransferase from Candida parapsilosis-Molecular cloning and characterization of purified recombinant enzymes. Eur. J. Biochem.

[18] Hegh I, Patkar S, Halkier T, Hansen MT (1995). Two lipases from Candida antarctica: Cloning and expression in *Aspergillus oryzae*. Can. J. Bot.

[19] Zhang JW, Zeng RY (2008). Molecular cloning and expression of a cold-adapted lipase gene from an Antarctic deep sea psychrotrophic bacterium Pseudomonas sp. 7323. Mar. Biotechnol.

[20] Santarossa G, Lafranconi PG, Alquati C, DeGioia L, Alberghina L, Fantucci P, Lotti M (2005). Mutations in the “lid” region affect chain length specificity and thermostability of a Pseudomonas fragi lipase. FEBS Lett.

[21] Zhang J, Lin S, Zeng RY (2007). Cloning, expression, and characterization of a cold-adapted lipase gene from an Antarctic deep-sea psychrotrophic bacterium, Psychrobacter sp. 7195. J. Microbiol. Biotechnol.

[22] Brady L, Brzozowski AM, Derewenda ZS, Dodson E, Dodson G, Tolley S, Turkenburg JP, Christiansen L, Hugejensen B, Norskov L, Thim L, Menge U (1990). A Serine protease triad forms the catalytic center of a triacylglycerol lipase. Nature.

[23] Tang SJ, Sun KH, Sun GH, Chang TY, Wu WL, Lee GC (2003). A transformation system for the nonuniversal CUG(Ser) codon usage species Candida rugosa. J. Microbiol. Methods.

[24] Tang SJ, Shaw JF, Sun KH, Sun GH, Chang TY, Lin CK, Lo YC, Lee GC (2001). Recombinant expression and characterization of the Candida rugosa lip4 lipase in Pichia pastoris: Comparison of glycosylation, activity, and stability. Arch. Biochem. Biophys.

[25] Tang SJ, Sun KH, Sun GH, Chang TY, Lee GC (2000). Recombinant expression of the Candida rugosa lip4 lipase in *Escherichia coli*. Protein Expr. Purif.

[26] Brocca S, Schmidt-Dannert C, Lotti M, Alberghina L, Schmid RD (1998). Design, total synthesis, and functional overexpression of the Candida rugosa lip1 gene coding for a major industrial lipase. Protein Sci.

[27] Chang SW, Lee GC, Shaw JF (2006). Efficient production of active recombinant Candida rugosa LIP3 lipase in *Pichia pastoris* and biochemical characterization of the purified enzyme. J. Agric. Food Chem.

[28] Pfeffer J, Richter S, Nieveler J, Hansen CE, Rhlid RB, Schmid RD, Rusnak M (2006). High yield expression of lipase a from Candida antarctica in the methylotrophic yeast *Pichia pastoris* and its purification and characterisation. Appl. Microbiol. Biotechnol.

[29] Gerday C, Aittaleb M, Arpigny JL, Baise E, Chessa JP, Garsoux G, Petrescu I, Feller G (1997). Psychrophilic enzymes: A thermodynamic challenge. Biochim. Biophys. Acta Protein Struct. Mol. Enzymol.

[30] Feller G, Narinx E, Arpigny JL, Aittaleb M, Baise E, Genicot S, Gerday C (1996). Enzymes from psychrophilic organisms. FEMS Microbiol. Rev.

[31] Choo DW, Kurihara T, Suzuki T, Soda K, Esaki N (1998). A cold-adapted lipase of an Alaskan psychrotroph, Pseudomonas sp. strain B11-1: Gene cloning and enzyme purification and characterization. Appl. Environ. Microbiol.

[32] Mayordomo I, Randez-Gil F, Prieto JA (2000). Isolation, purification, and characterization of a cold-active lipase from *Aspergillus nidulans*. J. Agric. Food Chem.

[33] Choi WC, Kim MH, Ro HS, Ryu SR, Oh TK, Lee JK (2005). Zinc in lipase L1 from *Geobacillus stearothermophilus* L1 and structural implications on thermal stability. FEBS Lett.

[34] Kiran GS, Shanmughapriya S, Jayalakshmi J, Selvin J, Gandhimathi R, Sivaramakrishnan S, Arunkumar M, Thangavelu T, Natarajaseenivasan K (2008). Optimization of extracellular psychrophilic alkaline lipase produced by marine Pseudomonas sp. (MSI057). Bioprocess Biosystems Eng.

[35] Dong H, Gao SJ, Han SP, Cao SG (1999). Purification and characterization of a Pseudomonas sp. lipase and its properties in non-aqueous media. Biotechnol. Appl. Biochem.

[36] Kojima Y, Shimizu S (2003). Purification and characterization of the lipase from Pseudomonas fluorescens HU380. J. Biosci. Bioeng.

[37] Shimada Y, Koga C, Sugihara A, Nagao T, Takada N, Tsunasawa S, Tominaga Y (1993). Purification and characterization of a novel solvent-tolerant lipase from *Fusarium heterosporum*. J. Ferment. Bioeng.

